# Screening of reference genes for expression analysis in the study of soldier caste differentiation of Formosan subterranean termite *Coptotermes formosanus* Shiraki

**DOI:** 10.7717/peerj.7981

**Published:** 2019-11-05

**Authors:** He Du, Wenjing Wu, Xueyi Huang, Zhiqiang Li

**Affiliations:** Guangdong Key Laboratory of Animal Conservation and Resource Utilization, Guangdong Public Laboratory of Wild Animal Conservation and Utilization, Guangdong Institute of Applied Biological Resources, Guangzhou, China

**Keywords:** Methoprene bioassay, Expression stability, Normalization

## Abstract

The Formosan subterranean termite, *Coptotermes formosanus* Shiraki, is a serious pest worldwide. Juvenile hormone analog (and its analogs such as methoprene) can induce the transformation of the worker caste into soldier caste in *C. formosanus*. However, several factors, such as feeding substrate and colony origin, influence the proportion of solider formation. The molecular mechanism of worker to soldier transformation of *C. formosanus* is still not clear. RT-qPCR is a powerful tool for molecular studies. Accurate gene quantification by the relative quantification method requires a stable expressed gene as the reference gene. However, no reference genes were available for this species in the methoprene bioassay. To study the problem of gene response to methoprene by RT-qPCR we have to first screen reference genes in *C. formosanus*. Workers were fed with methoprene. Termites were collected during the methoprene bioassay and separated into head and thorax+abdomen. Expression profiles of 10 candidate reference genes in the two body part types were investigated using RT-qPCR. The results were analyzed by a set of established methods (geNorm, NormFinder, BestKeeper, and RefFinder) as well as comparative ΔCt method. Our results suggest that RPS18 is the most stably-expressed gene both in the head and thorax+abdomen for expression analysis in the methoprene bioassay of *C. formosanus*. The screening of suitable reference genes in *C. formosanus* establishes the foundation for the molecular study of soldier caste differentiation in this species.

## Introduction

Termites are social insects, with the division of labor as one of its characteristics. The soldier caste is mainly responsible for the defense of the colony. Juvenile hormone (JH) and its analog, juvenile hormone analog (JHA) can induce superfluous soldiers within the colony (Lüscher, 1969; Hrdý & Křeček, 1972; Hrdý, 1973). The artificial induction of the soldier caste provides a basis for the molecular study of soldier caste differentiation. Based on the JH/JHA bioassay, our knowledge related to termite soldier differentiation was increased. For example, hexamerin was identified as a status quo-maintaining molecule to inhibit worker-to-soldier transformation (Zhou, Oi & Scharf, 2006). Cytochrome P450, the *Hox* gene and the IIS pathway were also involved in the development process (Cornette et al., 2006; Zhou et al., 2006; Toga, Saiki & Maekawa, 2013; Hattori et al., 2013). A recent study showed that Methoprene-tolerant (Met) protein is involved in the morphogenesis of *Zootermopsis nevadensis* (Hagen) soldier development (Masuoka et al., 2015). However, the molecular study of the Formosan subterranean termite, *Coptotermes formosanus* Shiraki, has fewer reports compared to those of other termite species. *C. formosanus* poses serious threats globally, and costs billions of dollars to control (Su, 1994). However, molecular interactions during metamorphosis from worker to soldier are still not clear. Investigation of the signaling cascade during solider development could offer a molecular control target in order to induce a high proportion of soldiers that could lead to the collapse of the colony.

The RT-qPCR is a powerful tool for quantification of gene expression. It is sensitive even to small differences in the quantity of RNA. The relative quantification method of RT-qPCR requires a reference gene to normalize the amount of RNA in the sample. Ideally, the reference gene should express stably in all experiment conditions. However, there are no universal reference genes. The reference gene used for each experiment is specific to the treatment of the experiment, and is screened for the specific experiment. Many candidate reference genes were tested for the specific purpose of the experiment in order to select the most stable one. Researches were conducted to screen suitable reference genes in different species (Yang et al., 2014, 2015; Zhao et al., 2014; Tan et al., 2015).

For the study of genes involved in soldier caste differentiation, several references genes were used for normalization of target genes in termites, such as *RPL13a* in *Z. nevadensis* (Masuoka et al., 2015) and β-actin in *Reticulitermes flavipes* (Kollar) (Zhou, Oi & Scharf, 2006). However, no reference gene was available for the JH/JHA bioassay in *C. formosanus*. The suggested reference genes used in other species may not be accurate for the molecular study of *C. formosanus*. As a result, the objective of this project is to investigate the expression stability of 10 genes to discover the most stably expressed gene in methoprene bioassay. A total of 10 constitutive genes were selected to test their suitability as the reference gene in the methoprene bioassay. Those candidate genes are essential for maintaining cellular functions. They are involved in gene expression and cellular respiration, and act as structural proteins. The genes engaged in gene expression include *60S ribosomal protein L32* (*RPL32*), *elongation factor 1-α* (*EF1-α*), *40S ribosomal protein S18* (*RPS18*), *60S ribosomal protein L13a* (*RPL13a*), *70 KDa heat shock protein* (*HSP70*), and *18S ribosomal RNA* (*18S*). The genes responsible for cellular respiration include *NADH dehydrogenase* (*NADH*), *glyceraldehyde 3-phosphate dehydrogenase* (*GAPDH*), *mitochondrial cytochrome b* (*Cyt-b*). Finally, β-*actin* is the structural protein gene selected for screening in this study.

## Materials and Methods

### Methoprene bioassay and sample preparation

Termites (*C. formosanus*) were collected using the method of Su & Scheffrahn (1986) from Dafushan Forest Park, Shangchong Fruit Tree Park and Sun Yat-sen University in Guangzhou city of China. No specific permissions were required for collecting *C. formosanus* termites. Termites were then kept in the laboratory and were used within 2 weeks.

Circles of filter paper (32 mm in diameter) were cut from a 60 by 60 cm filter paper (Double ring qualitative filter paper FAST, Hangzhou, China). An acetone solution of methoprene (90% in purity, Raw Material Medicine Reagent Co. Ltd., Nanjing, China) was dripped evenly onto the filter paper to make the compound as 1,000 ppm. All pieces of paper were dried for 15 min to evaporate the acetone. Two pieces of treated filter paper were put in the Petri dish. A volume of 230 μL deionized water was dripped evenly on the paired filter paper in each Petri dish. A total of 20 workers (undifferentiated larvae of at least the third instar) were then placed into the Petri dish (35 mm in diameter). The Petri dishes were placed into a plastic box with four pieces of wet paper towels to maintain the moisture. The boxes were kept in an incubator at 28 °C.

Samples from one colony were collected at seven different points: before feeding workers on filter paper (designated as “0D”), after feeding workers methoprene for 1, 4, 7, 10, and 13 days (also referred to as “1D, 4D, 7D, 10D, 13D”), and after pre-soldiers formed. Pre-soldiers usually appeared after feeding workers methoprene about 2 weeks and reached the plateau around 3 weeks. On 0D, 40 workers (20 workers × 2 groups) were collected. On 1D, 4D, 7D, 10D and 13D, two Petri dishes of workers (20 workers × 2 Petri dishes) were collected at each point. After pre-soldiers formed, only pre-soldiers from two Petri dishes were harvested. Therefore, 7 × 2 = 14 samples were collected for one colony. Four colonies were used.

Workers and pre-soldiers were decapitated with a scalpel. The head was put into a 1.5 mL Eppendorf tube, and the remaining thorax and abdomen (thorax+abdomen) were put into another 1.5 mL Eppendorf tube. The tubes were then immersed into liquid nitrogen and then stored at −80 °C until RNA extraction.

### RNA extraction and quantitation

RNA of head and thorax+abdomen was extracted using the TRIzol method. The concentration of RNA was measured with the TGem Spectrophotometer Plus (Tiangen, Beijing, China). The integrity of RNA was visualized by agarose gel electrophoresis. After confirmation of the purity and integrity of the RNA, RNA was transcribed into cDNA with FastKing RT Kit (with gDNase) (Tiangen, Beijing, China) following the manufacturer’s protocol.

The sequences of the following genes of *C. formosanus* (txid: 36987) were retrieved from NBCI: *NADH* subunit 5 (AB626145.1), *β-actin* (KC740712), *GAPDH* (KC740815), *RPL32* (KC632407), *Cyt-b* (AB626145.1), *EF1-α* (KC632472), *RPS18* (JK354152). The sequences of *RPL13a* and *HSP70* were acquired from the Sequence Read Archive accession SRP068272 (https://www.ncbi.nlm.nih.gov/sra/?term=SRP068272). The primer sequences of *18S* referred to Zhang et al. (2011). All the other primers were designed with Primer Premier 6.0. RT-qPCR efficiency for a primer was measured by using different concentrations of cDNA as a template.

The combination of ten primers and seven templates (cDNA of 0D, 1D, 4D, 7D, 10D, 13D workers, and pre-soldiers) from one replicate was tested in one RT-qPCR run, and 16 PCR runs (4 colonies × 2 body part types × 2 replicates) were implemented in total. Tiangen’s Talent qPCR PreMix (SYBR Green) was used to make the reaction system for RT-qPCR. All the ingredients were added according to the protocol’s recommendation to make a final volume of 25 μL (2 × Talent qPCR PreMix: 12.5 μL, F primer (10 µM): 0.75 μL, R primer (10 µM): 0.75 μL, cDNA: one μL, 50 × reference dye: 0.5 μL, RNase-Free ddH_2_O: 9.5 μL). Quantitative real-time PCR was performed with Stratagene Mx3000P. The program used for amplification was denaturation at 95 °C for 3 min, followed by 40 cycles of denaturation at 95 °C for 5 s, and annealing/extension at 60 °C for 15 s. To confirm the specificity of primers, the melting/dissociation curve analysis was performed at the end of the amplification. After the PCR run, the reaction mixture of the RT-qPCR was checked by agarose gel electrophoresis to examine the amplification results.

### Data analysis

The amplification efficiency, *E* (%), was calculated by the formula: *E* = 10^(−1/slope)^ −1. The slope was obtained from the regression line, which was generated by plotting the Ct value against the log cDNA copy number. In order to test the robustness of the candidate reference genes, linear mixed model was used to analyze Ct values. Body parts and feeding time were the main effects and colony as the mixed effect. Pairwise comparisons were conducted using Tukey’s HSD test if there were significant differences (α = 0.05). The *P*-values of the multiple comparisons were adjusted by the corrected method of Holm (1979). The statistical analysis was conducted using R 3.3.1 statistical (R Development Core Team, 2016). In order to examine the suitability of the ten genes as reference genes, the Ct values were analyzed by five methods: geNorm (Vandesompele et al., 2002), NormFinder (Andersen, Jensen & Ørntoft, 2004), BestKeeper (Pfaffl et al., 2004), ΔCt method (Silver et al., 2006), and RefFinder (Xie et al., 2012). Since the geNorm and NormFinder require transformed Ct values as their input value, the data was transformed as follows: ΔCt was calculated by subtracting the smallest Ct of all the samples of a gene from the Ct value, and 2^−ΔCt^ was then calculated. The transformed data (2^−ΔCt^) was then entered into geNorm and NormFinder, and the calculation was performed according to the manuals of the algorithms. The original Ct values were input into BestKeeper for analysis. In the comparative ΔCt method, the mean standard deviation was first calculated for each gene; then, the ranking of gene stability was achieved by sorting the mean SD from the smallest to the largest. The Ct values were also input into RefFinder to calculate a comprehensive stability value.

## Results

**Figure 1:**
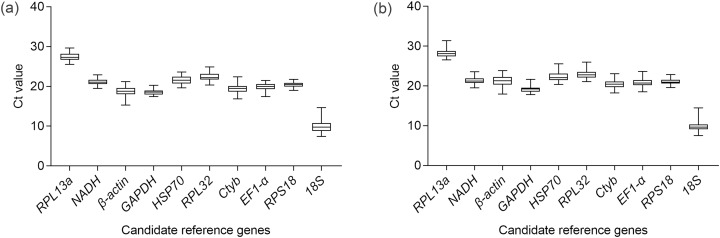
Expression profiles of 10 candidate reference genes in the methoprene bioassay of *C. formosanus*. The median is the line inside the box. The lower and upper edges of the box are the first and third quartile, respectively. The upper horizontal line is the maximum Ct value. The lower horizontal line is the minimum Ct value. (A) Expression profiles of the 10 candidate genes in the head. (B) Expression profiles of the 10 candidate reference genes in the thorax+abdomen.

The sequence and efficiency of the primers are shown in Table 1. The agarose gel demonstrated one amplicon band of desired length for each candidate reference gene. The dissociation curves of all the primers also showed one peak. Both the electrophoresis results and the dissociation curves confirmed the specificity of the primers. Among all the tested genes, *RPL13a* had the lowest expression level as demonstrated by its highest Ct value. On the contrary, the gene with the highest expression level was *18S* (Fig. 1). Feeding time influenced the Ct values of *β-actin* (χ^2^ = 30.53, d*f* = 5, *P* < 0.001), *GAPDH* (χ^2^ = 41.66, d*f* = 5, *P* < 0.001), *HSP70* (χ^2^ = 21.60, d*f* = 5, *P* < 0.001), *RPL32* (χ^2^ = 28.75, d*f* = 5, *P* < 0.001), *EF1α* (χ^2^ = 22.30, d*f* = 5, *P* < 0.001), *RPS18* (χ^2^ = 20.55, d*f* = 5, *P* < 0.001) (Table S1). The detailed results of multiple comparison of Ct values on different feeding time were shown in Table S2. Body parts had effects on the Ct values of *RPL13a* (χ^2^ = 7.58, d*f* = 1, *P* = 0.006), *β-actin* (χ^2^ = 109.22, d*f* = 1, *P* < 0.001), *GAPDH* (χ^2^ = 18.08, d*f* = 1, *P* < 0.001), *HSP70* (χ^2^ = 4.11, d*f* = 1, *P* = 0.04), *Ctyb* (χ^2^ = 10.44, d*f* = 1, *P* = 0.001), *EF1-α* (χ^2^ = 10.10, d*f* = 1, *P* = 0.001) and *RPS18* (χ^2^ = 10.33, d*f* = 1, *P* = 0.001) (Table S1). However, after comparison between Ct values of head and of thorax+abdoman for different feeding time, no significant difference was observed for *HSP70* (Table S3). There was an interaction effect between body parts and feeding time on the Ct values of *NADH* (χ^2^ = 13.34, d*f* = 5, *P* = 0.02), *GAPDH* (χ^2^ = 16.20, d*f* = 5, *P* = 0.006) and *Ctyb* (χ^2^ = 12.84, d*f* = 5, *P* = 0.02). *18S* was the only gene whose Ct values were not influenced by the main effects and the interaction effect (Table S1). The analysis results of the five algorithms were presented separately for the head and the thorax+abdomen as follows.

**Table 1:** Primers of 10 candidate reference genes used for qPCR in the methoprene bioassay of *C. formosanus*.

Gene		Primer sequence	Amplicon length (bp)	*E* (%)	*R*^2^
*RPL13a*	F	TGCCCAGGTTTGTCTCTTATTC	143	102.9	0.9993
	R	CTACCACTCCCAATACACCATTC			
*NADH*	F	TTGGGTTGGGATGGTTTAGG	117	96.7	0.9944
	R	AGCCACATCACCAATACGATTA			
*β-actin*	F	GACATCAAAGAGAAACTGTGCTATG	105	97.0	0.9958
	R	ACCATCAGGCAACTCGTATG			
*GAPDH*	F	GTGTGCCAGTTTCCAATGTG	101	96.7	0.9960
	R	TGCTGCTGCTTCCTTAACT			
*HSP70*	F	TCCTCTTTCGCTGGGTATTG	118	93.3	0.9955
	R	TGGCTGGTTGTCTGAGTATG			
*RPL32*	F	ATGGAGGAAACCTAAGGGTATTG	120	96.8	0.9910
	R	AAGCCAGTAGGAAGCATGTG			
*Cty-b*	F	GTATGAGGAGGATTCGCTGTAG	107	86.6	0.9898
	R	AATAGTAGGTGGACTAGGGTTATTG			
*EF1-α*	F	ATTGAGCGTAAGGAGGGTAAAG	112	92.0	0.9990
	R	CATCCTGAAGAGGAAGACGAAG			
*RPS18*	F	CGATGGCAAGAGGAAGGTTAT	107	99.6	0.9940
	R	CCCGCTTATCCAGATCAATGT			
*18S*	F	CGAGATTGAGCAATAACAGGTC	207	93.8	0.9732
	R	ACGTAATCAACGCGAGCTTATG			

### Analysis results for the head

**Figure 2:**
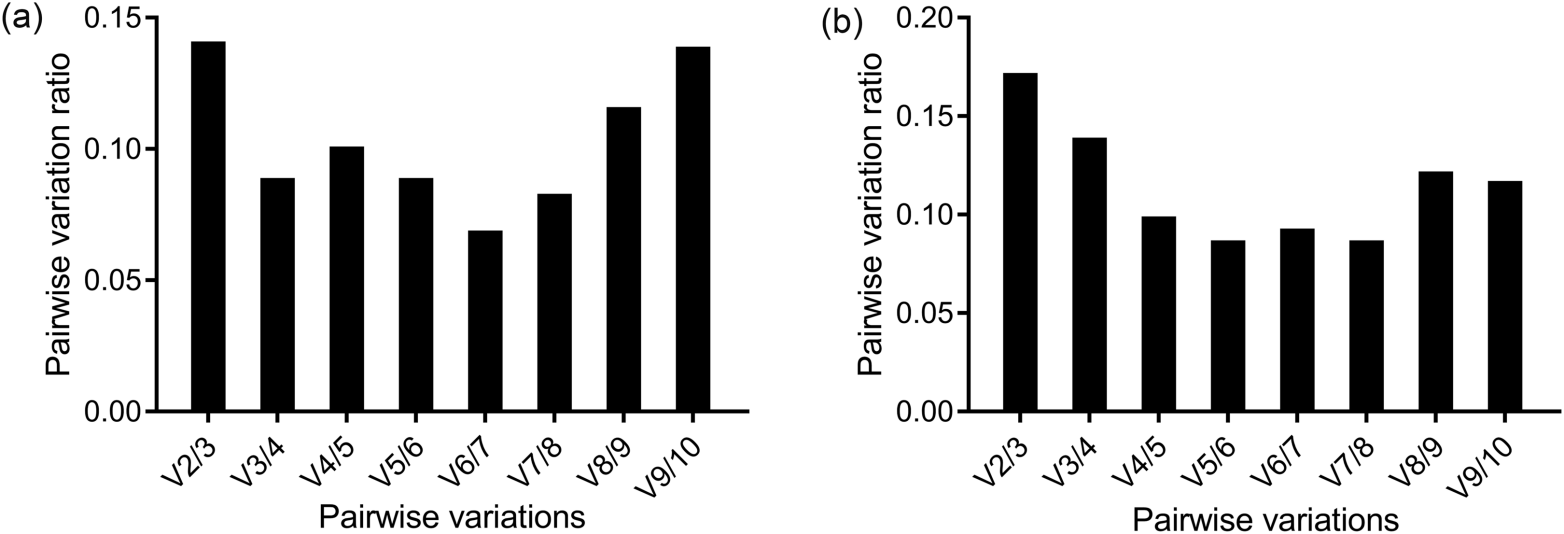
Determination of the optimal number of reference genes for normalization by geNorm in the methoprene bioassay of *C. formosanus*. The pairwise variations between two sequential normalization factors were used to determine the number of the optimal reference genes. The 0.15 threshold was used as a cut-off line. The pairwise variation in the head (A) showed that *V*_2/3_ = 0.141, so two genes were enough for an accurate normalization in the head. However, according to the pairwise variation in the thorax+abdomen (B), the second pairwise variation ratio was less than 0.15 (*V*_3/4_ = 0.139). So, in the thorax+abdomen, three genes were suggested to accurately normalize the target genes.

The ranking of the stability values of the 10 candidate reference genes for the head is presented in Table 2. The analysis results of geNorm generated two figures, one showed the stability value, *M*, and another showed the pairwise variation, *V*. The geNorm excluded the least stable gene in each calculation step and returned the two most stable genes as the final result. The two most stable genes were *EF1-α* and *RPS18* according to geNorm. The *V*_2/3_ was below the cut-off value 1.5, so two genes were enough for the normalization of the results (Fig. 2A). The third most stable gene was *RPL32* based on geNorm. The ranking of gene stability analyzed by Normfinder and comparative ΔCt method was the same: *RPS18* was the most stable gene. BestKeeper returned two results based on two criteria. *GAPDH* was the gene with the least expression variability based the SD, while *HSP70* was the most reliable gene based on the Pearson’s correlation coefficient. However, *RPS18* was the second most reliable gene based on both criteria. RefFinder, which gave a comprehensive result based on the four methods above, revealed that *RPS18* was the most appropriate reference gene for the methoprene bioassay.

**Table 2:** Ranking of the stability values of the 10 candidate reference genes based on five algorithms in the methoprene bioassay in the head of *C. formosanus*.

RefFinder		ΔCt		NormFinder		geNorm		BestKeeper				
Gene	GM	Genes	SV	Gene	SV	Gene	SV	Gene	SD	Gene	[*r*]	*P*-value
*RPS18*	1.19	*RPS18*	0.66	*RPS18*	0.19	*RPS18*	0.34	*GAPDH*	0.56	*HSP70*	0.94	0.001
*EF1-α*	2.59	*HSP70*	0.71	*HSP70*	0.33	*EF1-α*	0.34	*RPS18*	0.57	*RPS18*	0.94	0.001
*HSP70*	3.25	*EF1-α*	0.71	*EF1-α*	0.36	*RPL32*	0.415	*NADH*	0.66	*EF1-α*	0.89	0.001
*RPL32*	4.12	*RPL32*	0.73	*RPL32*	0.40	*HSP70*	0.423	*RPL13a*	0.76	*RPL32*	0.89	0.001
*GAPDH*	4.30	*RPL13a*	0.74	*RPL13a*	0.42	*RPL13a*	0.48	*EF1-α*	0.77	*Ctyb*	0.86	0.001
*RPL13a*	4.73	*NADH*	0.74	*NADH*	0.43	*NADH*	0.52	*RPL32*	0.81	*RPL13a*	0.85	0.001
*NADH*	5.05	*GAPDH*	0.77	*GAPDH*	0.48	*GAPDH*	0.54	*HSP70*	0.87	*NADH*	0.82	0.001
*Ctyb*	8.00	*Ctyb*	0.89	*Ctyb*	0.66	*Ctyb*	0.59	*Ctyb*	0.98	*GAPDH*	0.78	0.001
*β-actin*	9.00	*β-actin*	1.19	*β-actin*	1.04	*β-actin*	0.71	*β-actin*	1.02	*β-actin*	0.66	0.001
*18S*	10.00	*18S*	1.47	*18S*	1.38	*18S*	0.86	*18S*	1.14	*18S*	0.60	0.001

**Notes:**

GM, geometric mean; SV, stability value; SD, standard deviation; [*r*], Pearson’s correlation coefficient.

### Analysis results for the thorax+abdomen

The screening of the reference genes for thorax+abdomen differed slightly from the head (Table 3). *V*_2/3_ = 0.172 was more than the 0.15 threshold as shown by the pairwise variation of geNorm. Two genes were not enough to achieve a reliable normalization. The *V*_3/4_ = 0.139, so three genes were required for a reliable normalization (Fig. 2B). Although the number of genes required for reliable normalization was different from that of the head according to geNorm, the genes with the highest stability were the same as that in the head; that is, *RPS18* and *EF1-α* were ranked as the most reliable. Both ΔCt method and Normfinder recognized *RPL32* as the most suitable gene for normalization, followed by *RPS18* as the second most stable gene. According to Bestkeeper, *RPS18* was revealed as the most suitable gene based on the SD criteria, while *RPL32* was the gene with the highest stability value according to the Pearson’s correlation coefficient. Finally, RefFinder suggested *RPS18* as the most suitable reference gene for normalization of RT-qPCR data.

**Table 3:** Ranking of the stability values of the 10 candidate reference genes based on five algorithms in the methoprene bioassay in the thorax+abdomen of *C. formosanus*.

RefFinder		ΔCt		NormFinder		geNorm		BestKeeper				
Genes	GM	Genes	SV	Genes	SV	Genes	SV	Genes	SD	Genes	[*r*]	*P*-value
*RPS18*	1.41	*RPL32*	0.73	*RPL32*	0.35	*RPS18*	0.45	*RPS18*	0.53	*RPL32*	0.92	0.001
*RPL32*	2.11	*RPS18*	0.78	*RPS18*	0.43	*EF1-α*	0.45	*GAPDH*	0.62	*HSP70*	0.91	0.001
*GAPDH*	3.08	*GAPDH*	0.78	*Ctyb*	0.45	*GAPDH*	0.52	*EF1-α*	0.727	*Ctyb*	0.90	0.001
*EF1-α*	3.22	*Ctyb*	0.78	*HSP70*	0.46	*RPL32*	0.58	*NADH*	0.728	*RPS18*	0.86	0.001
*Ctyb*	4.74	*HSP70*	0.79	*GAPDH*	0.47	*HSP70*	0.59	*RPL32*	0.81	*EF1-α*	0.856	0.001
*HSP70*	5.32	*EF1-α*	0.81	*EF1-α*	0.50	*Ctyb*	0.61	*RPL13a*	0.87	*GAPDH*	0.84	0.001
*NADH*	6.09	*NADH*	0.86	*NADH*	0.59	*NADH*	0.65	*Ctyb*	0.88	*RPL13a*	0.82	0.001
*RPL13a*	7.44	*RPL13a*	0.9	*RPL13a*	0.63	*RPL13a*	0.69	*HSP70*	0.94	*NADH*	0.78	0.001
*β-actin*	9.24	*β-actin*	1.24	*β-actin*	1.10	*β-actin*	0.80	*18S*	0.96	*18S*	0.70	0.001
*18S*	9.74	*18S*	1.29	*18S*	1.16	*18S*	0.90	*β-actin*	1.12	*β-actin*	0.69	0.001

**Notes:**

GM, geometric mean; SV, stability value; SD, standard deviation; [*r*], Pearson’s correlation coefficient.

## Discussion

According to RefFinder, *RPL18* was the most stable gene in the methoprene bioassay both in the head and thorax+abdomen. Therefore, we suggest that *RPL18* should be used for the normalization of gene expression in the methoprene bioassay. The development of the soldier caste is controlled by a gene regulatory network. During the transformation from worker to soldier, the JH titer increases, which activates JH receptor gene-*Met*, and the related signaling pathway (Masuoka et al., 2015). However, effort is still needed to reveal the mechanism therein. In this project, we identified the most stable gene from a gene set, which established the first step for the study of the molecular mechanism for the differentiation of the worker caste into the soldier caste.

Several transcriptomes of *C. formosanus* were published recently, which facilitated the selection of reference genes for our study (Hussain et al., 2013; Wu et al., 2016; Hussain, Wen & Tian, 2017). The sequences of the reference genes screened in our study were retrieved and confirmed by the transcriptome data. *RPL18* encodes the 60S ribosomal protein L18, which is a component of the 60S subunit of ribosome. The basic function of protein synthesis for the maintenance of life activities may explain the reason of its expression stability during the methoprene bioassay. Other genes, such as the *RPL32* and *EF1-α*, which are also involved in similar functions, can be used as reference genes. Another category of genes ranked in the top of the list were the genes involved in energy production, such as *NADH* and *GAPDH*. *NADH* encodes the NADH dehydrogenase and is involved in electron transport in mitochondria during oxidative phosphorylation. *GAPDH* encodes GAPDH and is involved in glycolysis. The genes of structural proteins, such as *β-actin*, are not recommended, as the genes are ranked near the end of the list generated by the algorithms. The gene that encodes α-tubulin was also tested in our preliminary experiment. However, the fluorescent signal did not even reach the threshold after 40 cycles in the head of *C. formosanus*, and the gene was not further tested. The *18S* showed the highest expression level among all the candidate genes. The median of the Ct values of 18S were 9.8 and 9.6 for the head and thorax+abdomen, respectively. The role of JH receptor in soldier caste formation is studied in *C. formosanus* in our laboratory currently. In our preliminary experiment, the Ct values of several target genes, such as the gene of hexmerin and Methoprene-tolerant protein, were around 20. Due to the large difference between the Ct value of *18S* and target genes, use of *18S* as normalization gene is also not recommended.

The analysis results of the four programs gave slightly different results. In comparison with other algorithms, which can calculate one most stable gene, geNorm returns at least the two most stable genes. geNorm ranks the genes according to the gene stability value, *M*, which is the arithmetic mean of pairwise variation of the gene with all the other reference genes. The number of reference genes needed for normalization lies in the pairwise variation ratio (*V*_*n*/*n*+1_). If the *V*_*n*/*n*+1_ ratio between two sequential normalization factors *NF_n_* and *NF*_*n*+1_ does not vary significantly, “n genes” alone are enough for reliable normalization. Vandesompele et al. (2002) suggested the 0.15 threshold as the cut-off value. So, in the head, inclusion of two genes was enough to meet the requirement of the 0.15 threshold. The analysis results of comparative ΔCt method, NormFinder and geNorm were consistent with each other and differed slightly with the BestKeeper results. According to BestKeeper, the SD value of *18S* and of *β-actin* in the head and the SD value of *β-actin* in the thorax+abdomen were more than one, which indicated expression variability of those genes. So, those genes were excluded from the suitable reference gene list. Although the ranking of best suitable reference gene varied among the algorithms, all the algorithms ranked *Ctyb*, *β-actin*, and *18S* as the least suitable genes.

Several house-keeping genes were chosen as the reference genes for the molecular study of soldier caste differentiation. For instance, *Cty-b* was used as the reference gene during the pyriproxyfen bioassay in the dampwood termite *Hodotermopsis sjostedti* Holmgren (Cornette et al., 2013). Masuoka et al. (2015) used *RPL13a* as the reference gene when they studied the function of *Met* and *Kr-h1* in the differentiation of soldier caste in *Z. nevadensis*. Zhou, Oi & Scharf (2006) analyzed three reference genes (β-*actin*, *HSP-70*, and *NADH*) using BestKeeper and NormFinder when studying the role of hexamerin in soldier differentiation induced by JH III, and used *β-actin* as the reference genes. Tian (2015) employed *HSP70* as the reference gene when studying the role of *Met* during soldier differentiation of *R. flavipes*. Even for the same species, utilization of reference genes could be specific to the purpose of the experiment. In *C. formosanus*, Tarver et al. (2014) suggested *18S* and *ribosomal protein L7* as the reliable genes, which was different from our results. The reason is that the objective of Tarver et al. (2014) was to compare gene expressions among different castes and body regions, in contrast to studying gene expression during the artificial induction of the soldier caste in our projects. Here, in our study, 10 genes were screened, some of which have not been tested in the JH or JHA bioassay, such as *GAPDH*.

Research on the Formosan subterranean termite, a worldwide pest, is mainly focused on its control. The study of the molecular mechanism of soldier differentiation lags behind that of other termite species, such as *R. flavipes* (Zhou, Oi & Scharf, 2006) and *Z. nevadensis* (Masuoka et al., 2015). Besides its economic importance, the Formosan subterranean termite is evolutionarily significant, as it is a transition species between the lower termites and higher termites. The response of *C. formosanus* to methoprene is influenced by a variety of factors, such as colony, testing matrix, and temperature (Park & Raina, 2003; Tarver et al., 2012). Molecular research on the Formosan subterranean termite could lead to thorough understanding of Isoptera. The screening of reference genes for the methoprene bioassay lays the foundation for further molecular study of this worldwide pest species, and may lead to new control methods for this species.

## Conclusions

A total of 10 genes were evaluated for their suitability as reference genes for expression analysis in methoprene bioassay of *C. formosanus*. We recommend that *RPS18* is used as the reference gene in both the head and thorax+abdomen. This research lays the foundation for the molecular study of the mechanism underlying soldier caste differentiation in *C. formosanus*.

## Supplemental Information

10.7717/peerj.7981/supp-1 Supplemental Information 1 Ct values of the candidate reference genes from the head samples. The first column is the name of the head samples. The first row is the name of the ten candidate reference genes. Each data point indicates the Ct value of a candidate reference gene from one sample. Four colonies were used. Each colony had two replicates. Each replicates had seven samples (referred to as 0D, 1D, 4D, 7D, 10D, 13D, and Pre).

10.7717/peerj.7981/supp-2 Supplemental Information 2 Ct values of the 10 candidate reference gens from the abdoman+thorax samples. The first column is the name of the abdoman+thorax samples. The first row is the name of the 10 candidate reference genes. Each data point indicates the Ct value of a candidate reference gene from one sample. Four colonies were used. Each colony had two replicates. Each replicates had seven samples (referred to as 0D, 1D, 4D, 7D, 10D, 13D, and Pre).

10.7717/peerj.7981/supp-3 Supplemental Information 3 Effects of body parts and feeding time on the Ct values of the 10 candidate reference genes.

## Additional Information and Declarations

### Data Availability

The following information was supplied regarding data availability: The raw measurements are available in Files S1 and S2.

## References

[ref-1] Andersen CL, Jensen JL, Ørntoft TF (2004). Normalization of real-time quantitative reverse transcription-PCR data: a model-based variance estimation approach to identify genes suited for normalization, applied to bladder and colon cancer data sets. Cancer Research.

[ref-2] Cornette R, Hayashi Y, Koshikawa S, Miura T (2013). Differential gene expression in response to juvenile hormone analog treatment in the damp-wood termite *Hodotermopsis sjostedti* (Isoptera, Archotermopsidae). Journal of Insect Physiology.

[ref-3] Cornette R, Koshikawa S, Hojo M, Matsumoto T, Miura T (2006). Caste-specific cytochrome P450 in the damp-wood termite *Hodotermopsis sjostedti* (Isoptera, Termopsidae). Insect Molecular Biology.

[ref-4] Hattori A, Sugime Y, Sasa C, Miyakawa H, Ishikawa Y, Miyazaki S, Okada Y, Cornette R, Lavine LC, Emlen DJ, Koshikawa S, Miura T (2013). Soldier morphogenesis in the damp-wood termite is regulated by the insulin signaling pathway. Journal of Experimental Zoology Part B: Molecular and Developmental Evolution.

[ref-6] Holm S (1979). A simple sequentially rejective multiple test procedure. Scandinavian Journal of Statistics.

[ref-7] Hrdý I (1973). Effect of juvenoids on termites and honeybees.

[ref-8] Hrdý I, Křeček J (1972). Development of superfluous soldiers induced by juvenile hormone analogues in the termite, *Reticulitermes lucifugus santonensis*. Insectes Sociaux.

[ref-9] Hussain A, Li Y-F, Cheng Y, Liu Y, Chen C-C, Wen SY (2013). Immune-related transcriptome of *Coptotermes formosanus* Shiraki workers: the defense mechanism. PLOS ONE.

[ref-10] Hussain A, Wen S-Y, Tian M-Y (2017). Exploring the caste-specific multi-layer defense mechanism of Formosan subterranean termites, *Coptotermes formosanus* Shiraki. International Journal of Molecular Sciences.

[ref-34] Lüscher M (1969). Die Bedeutung des Juvenilhormones für die Differenzierung der Soldaten bei der *Termite Kalotermes flavicollis*.

[ref-12] Masuoka Y, Yaguchi H, Suzuki R, Maekawa K (2015). Knockdown of the juvenile hormone receptor gene inhibits soldier-specific morphogenesis in the damp-wood termite *Zootermopsis nevadensis* (Isoptera: Archotermopsidae). Insect Biochemistry and Molecular Biology.

[ref-13] Park YI, Raina A (2003). Factors regulating caste differentiation in the Formosan subterranean termite with emphasis on soldier formation. Sociobiology.

[ref-14] Pfaffl MW, Tichopad A, Prgomet C, Neuvians TP (2004). Determination of stable housekeeping genes, differentially regulated target genes and sample integrity: BestKeeper – Excel-based tool using pair-wise correlations. Biotechnology Letters.

[ref-15] R Development Core Team (2016). R: a language and environment for statistical computing.

[ref-16] Silver N, Best S, Jiang J, Thein SL (2006). Selection of housekeeping genes for gene expression studies in human reticulocytes using real-time PCR. BMC Molecular Biology.

[ref-17] Su N-Y (1994). Field evaluation of a hexaflumuron bait for population suppression of subterranean termites (Isoptera: Rhinotermitidae). Journal of Economic Entomology.

[ref-18] Su N-Y, Scheffrahn RH (1986). A method to access, trap, and monitor field populations of the Formosan termite (Isoptera: Rhinotermitidae) in the urban environment. Sociobiology.

[ref-20] Tan Q-Q, Zhu L, Li Y, Liu W, Ma W-H, Lei C-L, Wang X-P (2015). A de novo transcriptome and valid reference genes for quantitative real-time PCR in *Colaphellus bowringi*. PLOS ONE.

[ref-21] Tarver MR, Florane CB, Zhang D, Grimm C, Lax AR (2012). Methoprene and temperature effects on caste differentiation and protein composition in the Formosan subterranean termite, *Coptotermes formosanus*. Journal of Insect Science.

[ref-22] Tarver MR, Mattison C, Florane CB, Hinchliffe DJ, Zhang D, Lax AR (2014). Screening of multiple potential control genes for use in caste and body region comparisons using RT-qPCR in *Coptotermes formosanus*. Sociobiology.

[ref-23] Tian L (2015). New Insights into the function and development of the soldier caste in termites.

[ref-24] Toga K, Saiki R, Maekawa K (2013). *Hox* gene deformed is likely involved in mandibular regression during presoldier differentiation in the nasute termite *Nasutitermes takasagoensis*. Journal of Experimental Zoology Part B: Molecular and Developmental Evolution.

[ref-25] Vandesompele J, De Preter K, Pattyn F, Poppe B, Van Roy N, De Paepe A, Speleman F (2002). Accurate normalization of real-time quantitative RT-PCR data by geometric averaging of multiple internal control genes. Genome Biology.

[ref-26] Wu W, Li Z, Zhang S, Ke Y, Hou Y (2016). Transcriptome response to elevated atmospheric CO_2_ concentration in the Formosan subterranean termite, *Coptotermes formosanus* Shiraki (Isoptera: Rhinotermitidae). PeerJ.

[ref-27] Xie F, Xiao P, Chen D, Xu L, Zhang B (2012). miRDeepFinder: a miRNA analysis tool for deep sequencing of plant small RNAs. Plant Molecular Biology.

[ref-28] Yang C, Pan H, Liu Y, Zhou X (2014). Selection of reference genes for expression analysis using quantitative real-time PCR in the pea aphid, *Acyrthosiphon pisum* (Harris) (Hemiptera, Aphidiae). PLOS ONE.

[ref-29] Yang C, Pan H, Noland JE, Zhang D, Zhang Z, Liu Y, Zhou X (2015). Selection of reference genes for RT-qPCR analysis in a predatory biological control agent, *Coleomegilla maculata* (Coleoptera: Coccinellidae). Scientific Reports.

[ref-30] Zhang D, Lax AR, Bland JM, Allen AB (2011). Characterization of a new endogenous endo-β-1,4-glucanase of Formosan subterranean termite (*Coptotermes formosanus*). Insect Biochemistry and Molecular Biology.

[ref-31] Zhao Y, Chen M, Wang T, Sun L, Xu D, Yang H (2014). Selection of reference genes for qRT-PCR analysis of gene expression in sea cucumber *Apostichopus japonicus* during aestivation. Chinese Journal of Oceanology and Limnology.

[ref-32] Zhou X, Oi FM, Scharf ME (2006). Social exploitation of hexamerin: RNAi reveals a major caste-regulatory factor in termites. Proceedings of the National Academy of Sciences of the United States of America.

[ref-33] Zhou X, Song C, Grzymala TL, Oi FM, Scharf ME (2006). Juvenile hormone and colony conditions differentially influence cytochrome P450 gene expression in the termite *Reticulitermes flavipes*. Insect Molecular Biology.

