# Supplementary material for: Screening of reference genes for expression analysis in the study of soldier caste differentiation of Formosan subterranean termite Coptotermes formosanus Shiraki
Source: PeerJ. 2019 Nov 5;7:e7981. doi: 10.7717/peerj.7981 (PMC6839520; doi:10.7717/peerj.7981)
Supplement: Supplemental Information 3 [file peerj-07-7981-s003.docx]

| **Gene** | **Source** | ***χ^2^*** | ***df*** | ***P* value** |
| --- | --- | --- | --- | --- |
| *RPL13a* | Body Parts | 7.5804 | 1 | 0.005901 ** |
|  | Feeding Time | 9.0982 | 5 | 0.105212 |
|  | Interaction | 6.2980 | 5 | 0.278289 |
| *NADH* | Body Parts | 0.0473 | 1 | 0.82786 |
|  | Feeding Time | 3.2583 | 5 | 0.66023 |
|  | Interaction | 13.3367 | 5 | 0.02042 * |
| *β-actin* | Body Parts | 109.2240 | 1 | < 2.2e-16 *** |
|  | Feeding Time | 30.5258 | 5 | 1.162e-05 *** |
|  | Interaction | 9.3729 | 5 | 0.09508 |
| *GAPDH* | Body Parts | 18.084 | 1 | 2.114e-05 *** |
|  | Feeding Time | 41.662 | 5 | 6.895e-08 *** |
|  | Interaction | 16.195 | 5 | 0.006308 ** |
| *HSP70* | Body Parts | 4.1149 | 1 | 0.0425075 * |
|  | Feeding Time | 21.5974 | 5 | 0.0006244 *** |
|  | Interaction | 5.1259 | 5 | 0.4007099 |
| *RPL32* | Body Parts | 0.0099 | 1 | 0.9208 |
|  | Feeding Time | 28.7470 | 5 | 2.599e-05 *** |
|  | Interaction | 3.5684 | 5 | 0.6131 |
| *Ctyb* | Body Parts | 10.4447 | 1 | 0.00123 ** |
|  | Feeding Time | 3.1351 | 5 | 0.67916 |
|  | Interaction | 12.837 | 5 | 0.02496 * |
| *EF1-α* | Body Parts | 10.0936 | 1 | 0.0014879 ** |
|  | Feeding Time | 22.2985 | 5 | 0.0004594 *** |
|  | Interaction | 3.3592 | 5 | 0.6447911 |
| *RPS18* | Body Parts | 10.3276 | 1 | 0.0013105 ** |
|  | Feeding Time | 20.5512 | 5 | 0.0009844 *** |
|  | Interaction | 2.8112 | 5 | 0.7290695 |
| *18S* | Body Parts | 1.7800 | 1 | 0.1822 |
|  | Feeding Time | 9.1342 | 5 | 0.1038 |
|  | Interaction | 2.4018 | 5 | 0.7912 |

Suppl. Table 1. Effects of the body parts and feeding time on the Ct values of the ten candidate reference genes.

Suppl. Table 2. Summary of *Q* values for multiple comparisons of Ct values on different feeding time in the head (H) and thorax+abdoman (T).

| Test details | Body parts | *RPL13a* | *NADH* | *β-actin* | *GAPDH* | *HSP70* | *RPL32* | *Ctyb* | *EF1-α* | *RPS18* | *18S* |
| --- | --- | --- | --- | --- | --- | --- | --- | --- | --- | --- | --- |
| 0D - 10D | H | NA | 1.0000 | 0.0986 | 1.0000 | 0.1991 | 0.1124 | 1.0000 | 0.2698 | 0.4689 | NA |
| 0D - 13D | H | NA | 1.0000 | 1.0000 | 1.0000 | 0.0606 | 0.0280 | 1.0000 | 0.8044 | 0.8100 | NA |
| 0D - 1D | H | NA | 1.0000 | 1.0000 | 1.0000 | 1.0000 | 1.0000 | 1.0000 | 1.0000 | 1.0000 | NA |
| 0D - 4D | H | NA | 1.0000 | 0.0986 | 1.0000 | 1.0000 | 1.0000 | 1.0000 | 1.0000 | 1.0000 | NA |
| 0D - 7D | H | NA | 1.0000 | 0.0005 | 1.0000 | 1.0000 | 0.3810 | 1.0000 | 1.0000 | 1.0000 | NA |
| 10D - 13D | H | NA | 1.0000 | 0.4155 | 1.0000 | 1.0000 | 1.0000 | 1.0000 | 1.0000 | 1.0000 | NA |
| 10D - 1D | H | NA | 1.0000 | 1.0000 | 1.0000 | 0.0606 | 0.0065 | 1.0000 | 0.0028 | 0.0061 | NA |
| 10D - 4D | H | NA | 1.0000 | 1.0000 | 1.0000 | 0.7101 | 1.0000 | 1.0000 | 0.4454 | 1.0000 | NA |
| 10D - 7D | H | NA | 1.0000 | 1.0000 | 1.0000 | 1.0000 | 1.0000 | 1.0000 | 1.0000 | 1.0000 | NA |
| 13D - 1D | H | NA | 1.0000 | 1.0000 | 1.0000 | 0.0156 | 0.0011 | 1.0000 | 0.0155 | 0.0138 | NA |
| 13D - 4D | H | NA | 1.0000 | 0.4060 | 1.0000 | 0.2440 | 0.6942 | 1.0000 | 1.0000 | 1.0000 | NA |
| 13D - 7D | H | NA | 1.0000 | 0.0040 | 1.0000 | 1.0000 | 1.0000 | 1.0000 | 1.0000 | 1.0000 | NA |
| 1D - 4D | H | NA | 1.0000 | 1.0000 | 1.0000 | 1.0000 | 0.4240 | 1.0000 | 1.0000 | 1.0000 | NA |
| 1D - 7D | H | NA | 1.0000 | 0.0986 | 1.0000 | 0.8255 | 0.0280 | 1.0000 | 0.1728 | 0.1757 | NA |
| 4D - 7D | H | NA | 1.0000 | 1.0000 | 1.0000 | 1.0000 | 1.0000 | 1.0000 | 1.0000 | 1.0000 | NA |
| 0D - 10D | T | NA | 0.6694 | 0.0986 | 0.0002 | 0.1991 | 0.1124 | 0.6370 | 0.2698 | 0.4689 | NA |
| 0D - 13D | T | NA | 1.0000 | 1.0000 | 0.0001 | 0.0606 | 0.0280 | 0.4811 | 0.8044 | 0.8100 | NA |
| 0D - 1D | T | NA | 1.0000 | 1.0000 | 1.0000 | 1.0000 | 1.0000 | 1.0000 | 1.0000 | 1.0000 | NA |
| 0D - 4D | T | NA | 1.0000 | 0.0986 | 0.3109 | 1.0000 | 1.0000 | 1.0000 | 1.0000 | 1.0000 | NA |
| 0D - 7D | T | NA | 1.0000 | 0.0005 | 0.0102 | 1.0000 | 0.3810 | 1.0000 | 1.0000 | 1.0000 | NA |
| 10D - 13D | T | NA | 1.0000 | 0.4155 | 1.0000 | 1.0000 | 1.0000 | 1.0000 | 1.0000 | 1.0000 | NA |
| 10D - 1D | T | NA | 0.7844 | 1.0000 | 0.0001 | 0.0606 | 0.0065 | 0.4919 | 0.0028 | 0.0061 | NA |
| 10D - 4D | T | NA | 1.0000 | 1.0000 | 0.4588 | 0.7101 | 1.0000 | 1.0000 | 0.4454 | 1.0000 | NA |
| 10D - 7D | T | NA | 1.0000 | 1.0000 | 1.0000 | 1.0000 | 1.0000 | 1.0000 | 1.0000 | 1.0000 | NA |
| 13D - 1D | T | NA | 1.0000 | 1.0000 | <0.0001 | 0.0156 | 0.0011 | 0.3663 | 0.0155 | 0.0138 | NA |
| 13D - 4D | T | NA | 1.0000 | 0.4060 | 0.3109 | 0.2440 | 0.6942 | 1.0000 | 1.0000 | 1.0000 | NA |
| 13D - 7D | T | NA | 1.0000 | 0.0040 | 1.0000 | 1.0000 | 1.0000 | 1.0000 | 1.0000 | 1.0000 | NA |
| 1D - 4D | T | NA | 1.0000 | 1.0000 | 0.1983 | 1.0000 | 0.4240 | 1.0000 | 1.0000 | 1.0000 | NA |
| 1D - 7D | T | NA | 1.0000 | 0.0986 | 0.0057 | 0.8255 | 0.0280 | 1.0000 | 0.1728 | 0.1757 | NA |
| 4D - 7D | T | NA | 1.0000 | 1.0000 | 1.0000 | 1.0000 | 1.0000 | 1.0000 | 1.0000 | 1.0000 | NA |

Notes:

1. The value in each cell is the *Q* value, which was the adjusted *P* value acquired by the corrected method of Holm. The value in red means significant difference (α=0.05).

2. NA means no multiple comparison is conducted, as there are no feeding time effect and interaction effect.

3. Samples collected just before feeding workers filter paper were designated as “0D”; samples collected after feeding workers methoprene-treated filter paper for 1, 4, 7, 10, and 13 days were designated as “1D, 4D, 7D, 10D, 13D”.

Suppl. Table 3. Summary of *q* values for comparisons of Ct values between head and thorax+abdoman at different feeding time.

| Feeding time | *RPL13a* | *NADH* | *β-actin* | *GAPDH* | *HSP70* | *RPL32* | *Ctyb* | *EF1-α* | *RPS18* | *18S* |
| --- | --- | --- | --- | --- | --- | --- | --- | --- | --- | --- |
| 0D | 0.0483 | 0.6219 | <0.0001 | 0.0004 | 0.2942 | NA | 0.0134 | 0.0131 | 0.0119 | NA |
| 10D | 0.0483 | 0.6219 | <0.0001 | 1.0000 | 0.2942 | NA | 0.9403 | 0.0131 | 0.0119 | NA |
| 13D | 0.0483 | 0.1760 | <0.0001 | 1.0000 | 0.2942 | NA | 0.8589 | 0.0131 | 0.0119 | NA |
| 1D | 0.0483 | 0.8752 | <0.0001 | 0.0022 | 0.2942 | NA | 0.0905 | 0.0131 | 0.0119 | NA |
| 4D | 0.0483 | 0.6219 | <0.0001 | 0.2646 | 0.2942 | NA | 0.0905 | 0.0131 | 0.0119 | NA |
| 7D | 0.0483 | 0.8752 | <0.0001 | 0.4141 | 0.2942 | NA | 0.2887 | 0.0131 | 0.0119 | NA |

See notes for Suppl. Table 2.
